# A gene-level test for directional selection on gene expression

**DOI:** 10.1093/genetics/iyad060

**Published:** 2023-04-10

**Authors:** Laura L Colbran, Fabian C Ramos-Almodovar, Iain Mathieson

**Affiliations:** Department of Genetics, Perelman School of Medicine, University of Pennsylvania, Philadelphia, PA 19104, USA

**Keywords:** evolution, gene regulation, selection, quantitative genetics, human evolution

## Abstract

Most variants identified in human genome-wide association studies and scans for selection are noncoding. Interpretation of their effects and the way in which they contribute to phenotypic variation and adaptation in human populations is therefore limited by our understanding of gene regulation and the difficulty of confidently linking noncoding variants to genes. To overcome this, we developed a gene-wise test for population-specific selection based on combinations of regulatory variants. Specifically, we use the $Q_{X}$ statistic to test for polygenic selection on *cis*-regulatory variants based on whether the variance across populations in the predicted expression of a particular gene is higher than expected under neutrality. We then applied this approach to human data, testing for selection on 17,388 protein-coding genes in 26 populations from the Thousand Genomes Project. We identified 45 genes with significant evidence (FDR<0.1) for selection, including *FADS1*, *KHK*, *SULT1A2*, *ITGAM*, and several genes in the HLA region. We further confirm that these signals correspond to plausible population-level differences in predicted expression. While the small number of significant genes (0.2%) is consistent with most *cis*-regulatory variation evolving under genetic drift or stabilizing selection, it remains possible that there are effects not captured in this study. Our gene-level $Q_{X}$ score is independent of standard genomic tests for selection, and may therefore be useful in combination with traditional selection scans to specifically identify selection on regulatory variation. Overall, our results demonstrate the utility of combining population-level genomic data with functional data to understand the evolution of gene expression.

## Introduction

Natural selection is one process by which populations respond to their environment. Therefore, identifying phenotypes, genes and variants influenced by selection is an important aspect of understanding how organisms and populations adapt. In humans, a major focus of studies of selection has been the identification of population-specific adaptations. This is usually in the hopes of better understanding the mechanisms underlying the phenotype (Crawford *et al.* 2017; Ilardo *et al.* 2018; Simonson *et al.* 2010) and using that information to improve human health. However, linking genomic signals of selection to specific phenotypes and evolutionary pressures remains challenging. It is believed that changes in gene expression underlie most recent evolution (King and Wilson 1975; Corradin *et al.* 2016), and are, therefore, the most likely changes to underlie selection on complex traits. On the other hand, across species gene expression seems to largely be under stabilizing selection or evolving neutrally (Rohlfs *et al.* 2014; Chen *et al.* 2018; Signor and Nuzhdin 2018). While there are some genes with evidence for directional selection on expression (Blekhman *et al.* 2008), the overall extent to which selection plays a role in gene regulation, in general, remains poorly characterized (Price *et al.* 2022).

One approach to testing for selection on complex traits is to start with an observed trait difference, then to test whether that difference is greater than expected compared to the genetic difference (Whitlock 2008), and work backwards to understand the mechanism. The limitation of this approach is that it is difficult to account for the environmental component of the phenotypic variance. Another approach, typical for genome-wide scans for selection, is to identify individual outlier haplotypes based on allele frequency or linkage disequilibrium (LD) patterns, then work forward to understand which variant is the causal one, and what it might be influencing (Voight *et al.* 2006). Since variants rarely act in isolation, many traits are polygenic and any signals of selection on complex traits could therefore be spread across many variants across the genome. This can be identified by looking for enrichment of locus-specific selection signals (Field *et al.* 2016). Somewhat intermediate to these approaches, the $Q_{X}$ statistic (Berg and Coop 2014) tests for polygenic selection by using genome-wide association results to test for systematically divergent allele frequencies among all independent variants associated with a phenotype, in theory capturing only genetic contributions to the phenotypic variance. However, in practice, even this approach can be confounded due to poorly controlled population stratification in the underlying GWAS (Berg *et al.* 2019; Sohail *et al.* 2019). It remains unclear to what extent polygenic selection is relevant for human adaptation.

Most variants associated with complex traits through genome-wide association studies (and therefore those most likely to be subject to selection) are noncoding and likely operate through changes in gene expression. Therefore, directional polygenic selection on complex traits may involve directional selection on gene expression. We aim to test for selection on the expression of specific genes, reasoning that this phenotype might reflect the effects of natural selection more clearly than other complex traits. However, gene regulation is complicated, and mapping individual variants to their effects and genes is challenging (Benton *et al.* 2019; Gasperini *et al.* 2020). In parallel to the development of GWAS methodology, there has also been a proliferation of methods and data to associate variants with gene expression. Single-variant eQTL studies are common, however, genes often have multiple eQTL acting in concert to modulate expression. In addition, it is often prohibitively expensive to obtain the RNA-seq data needed to study expression directly in very large samples. Joint-tissue Imputation (JTI) is a machine-learning method that was developed to fill that gap by using expression and functional genomics data across dozens of tissues to predict gene expression based on combinations of genetic variants (Zhou *et al.* 2020). These models and similar ones can be used in a transcriptome-wide association study (TWAS) to identify gene-level associations with complex traits (Petty *et al.* 2019; Zhu and Zhou 2020), and we have used them to study predicted differences between ancient populations (Colbran *et al.* 2021). However, whether predicted differences between populations, in fact, reflect real differences in expression and if so whether they are the result of directional selection are still open questions.

The goal of our study is to use the $Q_{X}$ test with eQTL instead of GWAS data to test for population-specific directional selection on combinations of variants that are associated with expression of specific genes. Done genome-wide, this results in a gene-level test for selection on regulatory variation, which we believe will be more specific than polygenic selection scans on higher-level traits and more interpretable than variant-level scans. Overall, this work identifies dozens of genes whose regulation has been influenced by population-specific selection, and demonstrates the utility of incorporating functional data into genome-wide scans for selection.

## Materials and methods

### Regulatory variant selection

To select regulatory variants and effect sizes, we started with the published JTI gene expression models, which were trained in 49 tissues in version 8 of the Genotype-Tissue Expression project (GTEx) using all common variants (MAF>0.05 in GTEx) (Zhou *et al.* 2020). The training process included variant selection and quantification of independent linear effects of combinations of variants on expression of each gene in each tissue. We used these models to predict expression in Lymphoblastoid Cell Lines (LCLs) for 447 individuals from the 1,000 Genomes (1 kG) Project (Geuvadis; Lappalainen *et al.* 2013; The 1000 Genomes Project Consortium 2015). These individuals represented 4 populations of European ancestry (GBR, FIN, CEU, TSI) and one of African ancestry (YRI). We calculated TPM for ENA project PRJEB3366 using EMBL-EBI’s REST API (accessed March 8, 2022). We compared the predicted to observed expression for these individuals by calculating a Spearman rank correlation for 7,251 gene models trained in LCLs across all individuals. We compared the agreement of the models to the variance explained by the models in the training data by calculating the rank correlation across all genes between the model $R^{2}$ and the predicted/observed correlation. For each gene, we selected the “best” model by choosing the model with the highest $R^{2}$ across all tissues. This resulted in 26,878 genes for which we could compare observed and predicted expression. For most other analyses, we restricted to 17,388 protein-coding genes.

### Qx score adaptation

We obtained effect sizes for variants by filtering the best models to include only protein-coding genes (based on the “protein_coding” annotation in GenCode v26). The resulting 17,388 genes had a median of 12 (maximum 101) variants with effect sizes to calculate $Q_{X}$. We calculated $Q_{X}$ as described by Berg and Coop (2014), using the effect sizes from each gene expression model in place of GWAS effect sizes. More specifically, $Q_{X}$ for each gene is calculated using their equation 10:


$$Q_{X}=\frac{{\vec{Z^{\prime }}}^{T}F^{-1}\vec{Z^{\prime }}}{2V_{A}}$$

$\vec{Z^{\prime }}$ is the transformation (mean centering and dropping the *m*th entry) of the vector of mean genetic values for *M* populations. The entries of $\vec{Z^{\prime }}$ are equal to $z_{k}^{\prime }=z_{k}-\frac{1}{M}\sum_{m=1}^{M}z_{m}$ for k=1,…,M−1 where the untransformed genetic values


$$z_{m}=2\sum_{l=1}^{L}\alpha_{l}p_{ml}$$

In our case, $\alpha_{l}$ is the effect size of the *l*th variant in the JTI model, while $p_{ml}$ is the frequency of that variant in the *m*th population. *F* is the (M−1)×(M−1) matrix describing the expected neutral covariance across populations, calculated using frequencies of 100 matched variants for each variant in the JTI model. We matched these variants by binning all variants into 25 bins based on alternate allele frequency in GTEx (i.e. a bin size of 0.02). $V_{A}$ is a scaling factor defined as


$$V_{A}=2\sum_{l=1}^{L}\alpha_{l}^{2}\epsilon_{l}(1-\epsilon_{l})$$

where $\epsilon_{l}$ is the mean frequency of the *l*th variant in the model across all *M* populations.

We calculated a per gene $Q_{X}$ score for 2,504 individuals from 26 populations from the high coverage hg38 1 kG data (The 1000 Genomes Project Consortium 2015; Byrska-Bishop *et al.* 2022) by using the effect sizes from the corresponding JTI model in place of the GWAS associations, and repeated our analyses in 929 individuals from 7 continental groups from the Human Genome Diversity Panel (HGDP) (Bergström *et al.* 2020). We plotted Manhattan plots with OpenMendel (Zhou *et al.* 2020).

### *P*-value calculation

While $Q_{X}$ was designed to be a test for polygenic selection testing genome-wide, independent sets of variants, our adaptation of it would be better described as a multivariant test for selection. The set of possible variants for each gene was pruned for LD at $r^{2}>0.8$ before the JTI models were trained (Zhou *et al.* 2020), and models are built around independent, additive effects for the variants ultimately included. However, these variants are much closer together (within 2 Mb), and models often include variants in moderate LD ($r^{2}\approx 0.4$) with each other. This means that, while the effect sizes are independent, the allele frequencies are not necessarily, and the degree to which the frequencies are correlated with each other varies across genes. This makes calculating a *P*-value for gene-level $Q_{X}$ statistics complicated.

We tried three different methods for calculating *P*-values (available in Supplementary Table S1). The first is the method used in the original $Q_{X}$ study, wherein we construct a “null” distribution of $Q_{X}$ scores for each gene by permuting the allele frequencies of the variants in the model (abbreviated as “freqPerm”; Supplementary Fig. S1). For each permutation, we drew a random frequency for each variant in the model, holding effect sizes constant, and repeated that 100,000 times (up to 1,000,000 times if $P<10^{-4}$). As expected, because this permutation strategy breaks the LD structure present in many gene models, the resulting *P*-values are extremely poorly calibrated (Supplementary Fig. S2).

We, therefore, calculated “corrected” *P*-values by instead fitting a gamma distribution to the $Q_{X}$ scores. These *P*-values are much less inflated (Fig. 2b), while the ordering of genes is highly correlated with the order the freqPerm *P*-values gave (Spearman ρ=0.993). They are, however, somewhat correlated with the technical characteristics of the gene models (Supplementary Fig. S2).

To control for the technical confounding, we also calculated *P*-values by permuting the effect sizes for each gene while holding allele frequencies of variants constant (abbreviated as “effPerm”). Specifically, we randomly sampled effect sizes from the distribution of all possible effect sizes in any model, while holding the effect direction for each variant constant. We sampled 100,000 times for each gene, up to 1,000,000 for those with $P<10^{-4}$. While this did control for the technical variables (Supplementary Fig. S3) and was still correlated with the corrected *P*-values (Spearman ρ=0.709), it has the side effect of narrowing the hypothesis we were testing. Rather than a broad test for selection on regulatory variants, this permutation scheme emphasizes coordinated differences between populations (i.e. in the same effect direction) across multiple variants in a model. This means that true selection on a single regulatory haplotype (e.g. in the case of *FADS1*) is not identified.

### Power calculations

We calculated the power of the gamma-corrected and effect-permuted test using simulations run in SLiM 4.0 (Haller and Messer 2022). Simulations begin with an “ancestral” population of 10,000 diploid individuals. For each individual, we simulated a 1 Mb “gene” which can accumulate eQTL mutations, along with a disjoint 100 kb neutral segment that can accumulate neutral mutations at the same rate as the gene. Effectively, these mimic the structure of the JTI models we use to characterize regulatory variants in real data. Expression of the simulated gene is under stabilizing selection, and eQTL mutation effect sizes are drawn from a standard normal distribution. The relative fitness of individuals is calculated from total eQTL mutation effect sizes. The ancestral population is allowed to reproduce for 20,000 generations, with a mutation rate of $8\times 10^{-9}$ and a recombination rate of $1\times 10^{-7}$. The fitness function is a Gaussian distribution function centered at mean 0 with a standard deviation 1.

After 20k generations, we split the ancestral population into 5 subpopulations of 10,000 individuals each (P1, P2, P3, P4, P5) and the simulations run for another 400 generations. After the ancestral population split, the mutation and recombination rates are lowered to $1\times 10^{-10}$ and $1\times 10^{-8}$, respectively, in order to simulate genes with similar numbers of regulatory variants as captured by the JTI models we are mimicking. We then applied directional selection to P5 by shifting the fitness optimum by a varying amount, while holding it constant for the other 4 populations. At the end of the simulations, we output the position, frequency, and effect size of the generated eQTL mutations and mutations from the neutral DNA segment. We ran 20k simulations (where each simulation represents 1 “gene”) for each of the 8 fitness optimum shift (FOS) conditions for P5 (FOS=0.01, 0.02, 0.05, 0.1, 0.2, 0.5, 1). For each simulation, we then calculated the $Q_{X}$ statistic and *P*-value using either the gamma correction or effect permutation (drawing effect sizes from either the neutral background or the other simulations in the same FOS). We set the significance threshold to $P<10^{-4}$.

### Population expression comparison

We predicted expression by applying the best JTI Models to the same individuals used to calculate $Q_{X}$ (2504 unrelated individuals from 1 kG, and 929 from HGDP). We summarized across populations by taking the median predicted expression within each population.

We also compared this predicted expression observed expression in LCLs for both datasets. For 1 kG, this was the same data described in *Regulatory Variant Selection*. For HGDP, this included 45 individuals from 5 geographic regions—Africa, the Middle East, East Asia, South Asia, and the Americas. We calculated the significance of the agreement across genes in the analysis as follows: For each gene, we calculated a Spearman correlation between the median observed and predicted expression across all populations. We then summed the correlations together. We calculated an empirical *P*-value for each plot by shuffling the medians 10,000 times and repeating the summation.

### Other selection scores

We obtained loss-of-function (LoF) tolerance scores for each gene from Gnomad v2 (Lek *et al.* 2016), and used the observed/expected number of LoF variants as a measure of conservation on the protein sequence (where a low score indicates more constraint). We downloaded the phyloP100way track from the UCSC Genome Browser, and calculated the average score for each gene across the 200 kb window centered around the gene using the bigWigAverageOverBed tool. Positive phyloP scores indicate greater conservation in the region.

We calculated iHS and nSL statistics in all 26 1 kG populations using SelScan 2.0.0 (Szpiech 2021). nSL was calculated using unphased genomes (Ferrer-Admetlla *et al.* 2014). For iHS (Voight *et al.* 2006), we used phased genomes, and polarized ancestral and derived alleles based on the chimpanzee reference genome panTro6. We interpolated recombination maps for our sites from 1 kG maps (Spence and Song 2022). For both, we focused on variants with MAF>0.05 in the population in question, then took the mean statistic across the 200 kb window around each gene.

### Enrichment tests

We tested for enrichment for particular gene sets by calculating a “tiered” enrichment. We sorted the genes by *P*-value, then tested for enrichment in the top *N* (for N=10, 20, 30, 50, 75, 100, 150, 200, 300, 500, 1,000, 2,000, 3,000, 5,000, and 10,000). Enrichment is calculated as the proportion of genes in the top *N* divided by the overall proportion that are in the gene set in question. We used the binomial test to calculate a *P*-value, and calculated a confidence interval for each *N* using the Agresti–Coull method (Agresti and Coull 1998). We did this for 3,788 housekeeping genes (Eisenberg and Levanon 2013) and for 2,899 LoF-intolerant genes, where a gene is LoF-intolerant if the upper bound of the 95% confidence interval of the observed/expected ratio is lower than 0.35 (Lek *et al.* 2016), as well as 5,352 genes that have undergone balancing selection on expression across species (Chen *et al.* 2018). We additionally tested sets of genes based on their function. These included 20 diet genes with previously identified signals of selection (Rees *et al.* 2020) as well as two sets of immune genes: 1,257 virus-interacting proteins (Enard *et al.* 2016) and 128 interferon response genes (all products in the gene ontology term GO:0032606 and all child terms).

## Results

### Testing for selection on regulatory variants

**Fig. 1.**
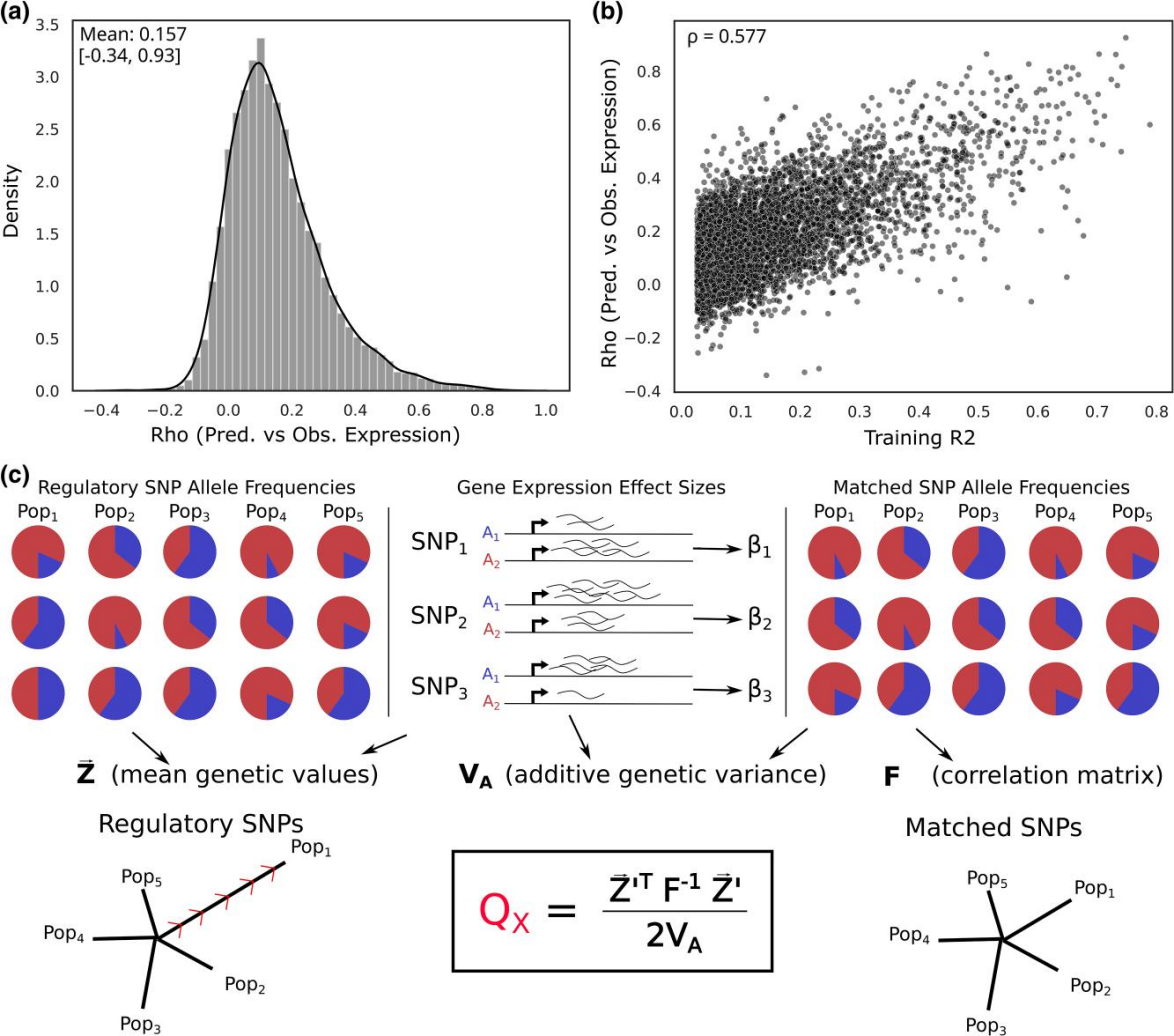
We adapted the Qx statistic to test for selection on regulatory variants. a) Spearman ρ between observed and predicted expression in 1 kG for 7,251 JTI models trained in GTEx LCLs, b) and that ρ plotted vs. the in-sample training $R^{2}$. c) Schematic of Qx calculation as applied to JTI models. The $Q_{X}$ score is based on the JTI effect sizes and allele frequencies across populations for the set of regulatory variants for each gene. The **F** matrix contains frequencies across the same populations for variants that were the same frequency in the JTI study population, but were not associated with expression of that gene, thereby modeling the expected covariance for these variants. $Q_{X}$ is higher when the regulatory variants for a gene exceed those expected patterns.

We identified a set of genetic variants that influence gene expression using published models of gene regulation built using JTI (Zhou *et al.* 2020), trained using the genotypes and transcriptomes from 49 tissues from the GTEx project (Aguet *et al.* 2017). Because these models borrow information across tissues, they are often correlated with each other, particularly for genes with shared regulatory patterns. We wanted to use one model per gene in order to limit the multiple-testing burden, since expression patterns across tissues are not independent. While ideally, we would like to study the most biologically relevant tissue for each gene, in general, that is not known. We, therefore, decided to focus on models that capture the most information about individual-level gene expression. We compared predicted expression to observed expression in LCLs for 5 populations from 1,000 Genomes (1 kG; N=447) (Lappalainen *et al.* 2013). We found that the rank correlation between predicted and actual expression for the gene models trained in GTEx LCLs was highly variable by gene (Fig. 1a; median Spearman ρ=0.12, maximum ρ=0.93). It was also significantly correlated with model performance during training (Fig. 1b; ρ with model $R^{2}=0.58$, $P=2.1\times 10^{-686}$), indicating that the training $R^{2}$ is a useful proxy for out-of-sample performance in tissues we have not measured directly. We, therefore, decided that for each gene we would use the model with the highest training $R^{2}$ (the “best model”), regardless of the primary tissue it was trained in. While we focused on these 26,878 best models (restricted to 17,388 protein-coding genes for most analyses), we suggest focusing on relevant tissues when testing specific hypotheses.

**Fig. 2.**
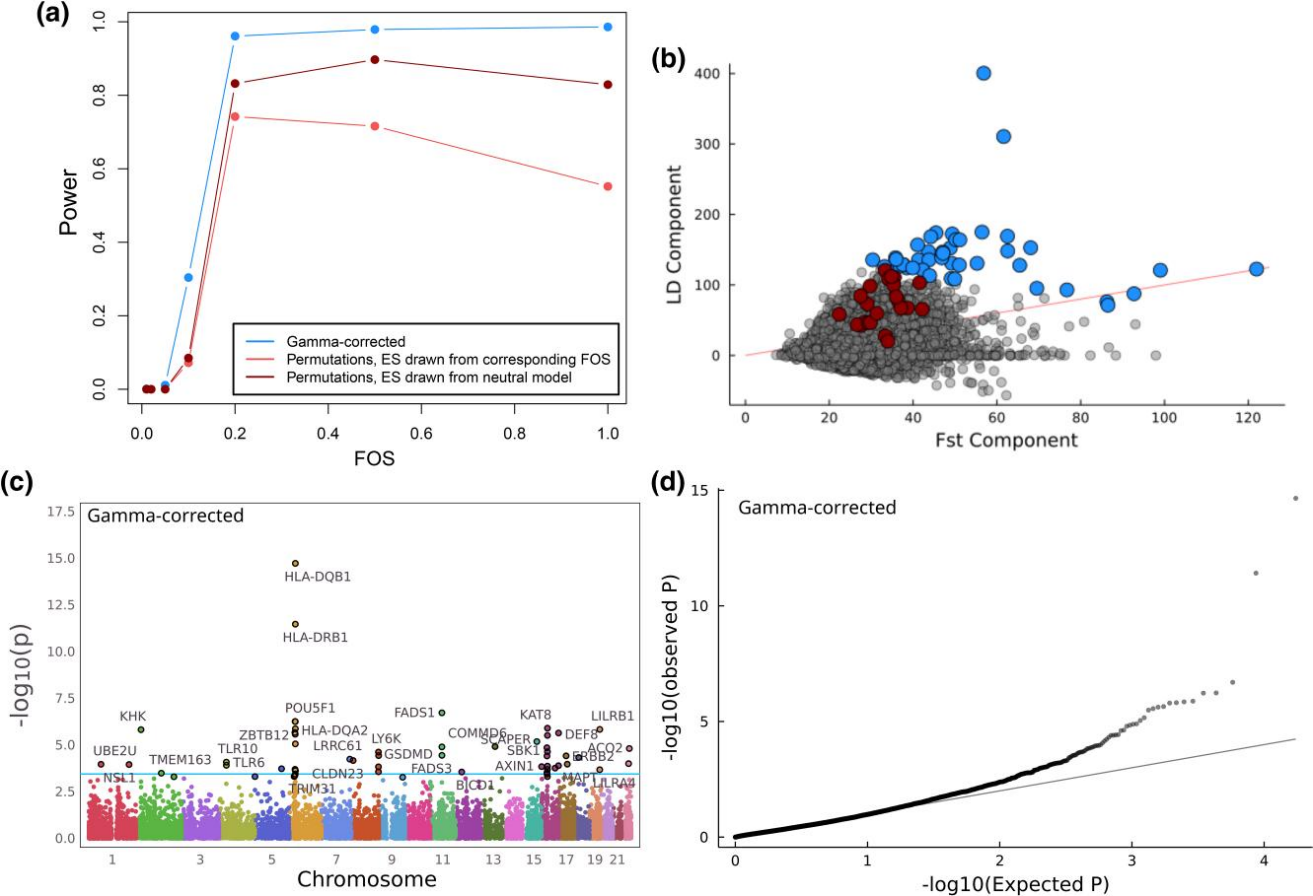
Using a gamma-corrected *P*-value, we identify 45 genes with evidence of selection. a) Power curves for each *P*-value method, based on simulations (Methods). We calculated the power for the gamma-corrected version of the $Q_{X}$ test, as well as for 2 variations of the effect-permuted test. In the first, we drew the effect sizes from the simulation that modeled the corresponding selection strength, and for the other from the neutral effect model. FOS, fitness optimum shift. b) The $Q_{X}$ score can be decomposed into its $F_{ST}$-like component and its LD-like component. Significant (FDR<0.1) genes in 1 kG for the gamma-controlled and effect-permuted *P*-values are highlighted in blue and red, respectively, while the red line indicates where $F_{ST}=LD$. The $Q_{X}$ score for each gene is obtained by adding the two components together. The Spearman rank correlation between the components is 0.15. c) Manhattan plot and d) QQ-plot for gamma-corrected $Q_{X}$  *P*-values for 17,388 genes. The horizontal line in (c) at −log_10_(p) = 3.95 corresponds to FDR<0.1.

To test for selection on gene regulation, we adapted the $Q_{X}$ test for polygenic selection (Berg and Coop 2014) (Fig. 1c). $Q_{X}$ was originally designed to test for excess variance in predicted phenotype across populations using loci associated with a polygenic trait and taking into account both allele frequencies and effect sizes of associated variants. Here, we test for excess variance among the set of regulatory variants that are correlated with expression of a gene, relying on frequencies and effect sizes as described by the JTI models. This allows us to test for overdispersion in genetic values of gene regulation (equivalently, coordinated differences in allele frequencies of regulatory variants; Methods). Theoretically, the $Q_{X}$ statistic follows a $\chi^{2}$ distribution with degrees of freedom one fewer than the number of populations under consideration (Supplementary Fig. S4). In practice, however, it can be over- or under-dispersed for reasons other than selection, such as population stratification or stabilizing selection, or if the allele frequencies do not follow a multivariate normal distribution.

One way to control for some of these effects is to calculate an empirical distribution. In Berg and Coop (2014), this was done by resampling allele frequencies for the variants in question genome-wide (Supplementary Fig. S1a). In our case, this procedure results in extremely poorly calibrated *P*-values (Supplementary Fig. S2); this is primarily because the gene regulation models break the assumption of independence between variants (discussed in more detail in Methods). While the effect sizes fit by the models are independent and variants were pruned for very high LD ($r^{2}>0.8$), most models still contain variants with lower levels of LD. Randomizing allele frequencies does not account for these residual correlations and does not produce well-calibrated *P*-values.

We, therefore, implemented two alternative strategies to compute *P*-values. First, instead of randomizing frequencies, we randomized effect sizes of the variants in each model by sampling from the distribution of effects across all models (Supplementary Fig. S1b). This tests specifically for coordination in the effect sizes of the variants conditional on allele frequencies. Second, we calculated empirical *P*-values by fitting a gamma distribution to the $Q_{X}$ distribution.

To evaluate these two approaches, we used SLiM (Haller and Messer 2022) to simulate varying degrees of population-specific selection for 20,000 genes with multiple regulatory variants, then calculated $Q_{X}$ and both gamma-corrected and effect-permuted *P*-values (Methods). We found that the gamma-corrected approach was uniformly more powerful than the permutation approach. Indeed, while the gamma-corrected test approaches a power of 1.0 under regimes with stronger selection, the effect-permuted version never reached that (Fig. 2a). We note that, while we parameterized the simulations such that the genes had similar numbers of regulatory variants to the JTI models used in the real data, we were not explicitly simulating all details of the models. These results are, therefore, only useful as an indication of the relative power of the approaches to each other, and not necessarily informative about the absolute power of the approach.

In order to understand the difference in power, we turned to the real data, noting that the $Q_{X}$ statistic can be decomposed into two components. The $F_{ST}$-like component captures allele frequency differences and the LD-like component incorporates the combinations of effect sizes and directions (Berg and Coop 2014). We performed this decomposition for the $Q_{X}$ scores calculated in 1 kG. Genes that are genome-wide significant (FDR<0.1) with gamma-corrected significant genes are outliers for both components. Genes that are significant with the permutation approach are not outliers in the $F_{ST}$ component and only slightly enriched in the LD component (Fig. 2b). In summary, the effect-permuted test is conservative, does not capture high-$Q_{X}$ outliers and does not identify genes known to have strong signals for selection such as *FADS1*, although it is less correlated with technical characteristics of the model such as the number of variants in a model and its training $R^{2}$ than the gamma-corrected approach (Supplementary Fig. S3). Due to the higher power, the rest of our analysis is based on the results of the gamma-corrected test.

In the 1 kG data, we identified 45 (out of 17,388) protein-coding genes with significant evidence of selection (FDR<0.1; Fig. 2c and d). Because the predicted expression of nearby genes shared regulatory haplotypes, these corresponded to 20 visible ‘peaks’ of nearby genes. These included several loci known to have experienced population-specific selection (e.g. *FADS1* and the TLR and HLA loci; Mathieson *et al.* 2015). These *P*-values are potentially still inflated by uncorrected population stratification, and are correlated with both the number of variants in a model as well as its training $R^{2}$ (Supplementary Fig. S3). These technical aspects of the model training should not necessarily influence patterns of selection, but probably do affect power. These characteristics make it difficult to identify which gene in a peak is most likely to be the one directly under selection, rather than merely influenced by the resulting allele frequency shifts. For comparison, when using the permutation test 23 genes have significant (FDR<0.1) *P*-values. Overall, the QQ plot shows little evidence of strong outliers (Supplementary Fig. S5). While there was no overlap in the significant genes in each *P*-value scheme, across all genes ordering was relatively highly correlated (Spearman ρ=0.71), suggesting the two methods capture some of the same information.

We also ran the $Q_{X}$ scan in HGDP in order to identify whether selection patterns and particular genes replicated in an independent dataset. For the gamma-corrected *P*-values, 48 genes were significant at FDR<0.1, and 4 peaks (HLA, *KHK*, *KAT8*, and *ACO2*) overlapped genes identified in 1 kG (Supplementary Fig. S6). For the effect size permuted *P*-values, no genes passed that significance threshold, though *SAMD10* (also identified in 1 kG) did have the smallest *P*-value. Overall, these results suggest that while some genes have experienced directional selection on expression driven by *cis*-regulatory variation, it is relatively rare.

### $Q_{X}$ is independent of other selection metrics

**Fig. 3.**
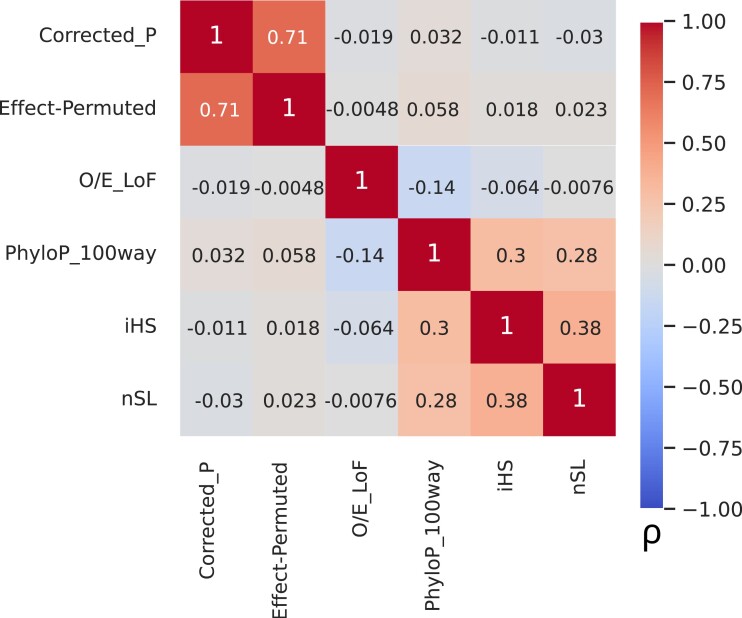
The $Q_{X}$ statistic is not correlated with other selection statistics. Pairwise heatmap of Spearman rank correlations between $Q_{X}$  *P*-values and various selection-related scores.

While our $Q_{X}$ analysis did identify several previously known genes, we wanted to know whether including gene regulation information was generally giving us more information than other, less-specific tests for selection. We, therefore, calculated the correlation between gene-level $Q_{X}$ and a variety of other measures of selection (Fig. 3). We find that the gamma-corrected *P*-values are not strongly correlated with either LoF intolerance or PhyloP, both of which are metrics of evolutionary constraint (Pollard *et al.* 2010; Lek *et al.* 2016), or with two haplotype-based tests for more recent selection (iHS, nSL, averaged across a window for each gene; highest Spearman ρ=−0.032 between *P* and PhyloP), although as expected these other four metrics do show some correlations with each other (highest Spearman ρ=0.38 between iHS and nSL). The same is true of the effect size permuted *P*-values.

We also tested whether different classes of genes were enriched for signals of directional regulatory selection. LoF-intolerant genes are somewhat depleted among the genes with the smallest gamma-corrected *P*-values (e.g. OR=0.281, P=0.0011 for the top 100 genes; Supplementary Fig. S7), suggesting that genes under strong constraint on their protein sequence also tend to be more constrained in their regulatory variation. Surprisingly, housekeeping genes, a broadly expressed class of genes responsible for basic cellular functions that we might expect to be similarly constrained, are somewhat enriched among genes with more evidence for selection (OR=2.64, $P=2.8\times 10^{-4}$ for top 500 gamma-corrected genes; OR=5.92, $P=1.5\times 10^{-3}$ for top 20 effect-permuted genes). This is consistent with patterns seen in our previous study of regulatory differences between ancient populations (Colbran *et al.* 2021), and may reflect reduced constraint in housekeeping promoter regions (Farré *et al.* 2007). We also tested for enrichment of genes of certain functional categories, such as viral-interacting proteins (Enard *et al.* 2016), immune genes that respond to interferon, and diet-related genes with known selection signals (Rees *et al.* 2020), as well as genes that have undergone stabilizing selection on expression across species (Chen *et al.* 2018), but found no significant trends for any of these categories for either set of *P*-values. Technically, the selected diet genes were significantly enriched among the top 10 gamma-corrected genes (P=0.021); however that signal is driven entirely by *FADS1*, and is therefore uninformative about broader patterns.

### Patterns of predicted expression among selected genes

**Fig. 4.**
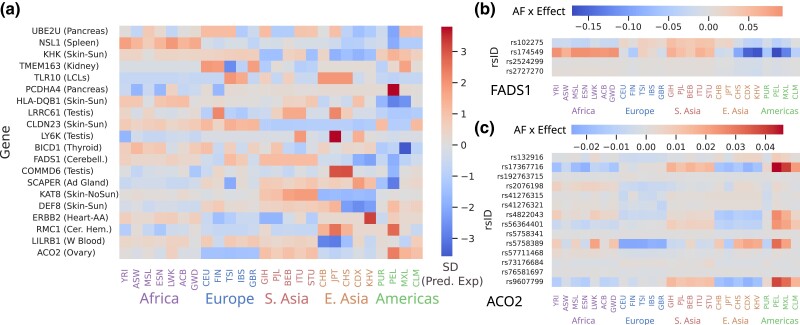
Different combinations of variant effects can drive predicted differences. a) Median predicted expression in each 1 kG population for the top gene in each peak of the gamma-corrected *P*-values. For display purposes, for each gene is standardized across populations. b) for *FADS1* there is one primary haplotype (tagged by rs174549), while for c) *ACO2* there are 3 variants driving the upregulation in PEL. Cells are colored by the product of JTI effect size times effect allele frequency in each population. Values in b) and c) are mean-centered for each variant.

We next wanted to determine what patterns of expression underlie the significant $Q_{X}$ scores. We, therefore, applied the best JTI models to predict expression in the same individuals we used in calculating the $Q_{X}$ scores and summarized these predictions across populations. As expected, we found that the genes showing significant selection differed between populations in predicted expression (Fig. 4a). For example, *LY6K* has a median predicted expression 3 standard deviations higher in Japanese populations than in most others. This is true for genes identified as significant under both *P*-value schemes (Supplementary Fig. S8), suggesting both methods identify genes with overall predicted differences. These predicted patterns are also largely similar in HGDP, despite the decreased resolution (Supplementary Fig. S9). As expected, these predicted differences do not always agree with patterns of observed expression in LCLs (P=0.241 for 1 kG); this could be because of tissue-specific expression patterns (we only have observed expression in LCLs), or because of differing environment or genetic background compared to the model training data (Supplementary Figs. S10 and S11).

While there are 20 peaks of significant selection in the gamma-corrected *P*-values calculated in 1 kG, it is unlikely that every gene in each peak is actually under selection. Instead, it is likely that the expression of one gene has an impact on fitness, and the expression of other genes with shared or linked regulatory variation hitchhikes along with the selected gene. For example, *FADS1* is a well-established example of a selected haplotype whose effect is correctly modeled and detected (tagged by rs174549; Fig. 4b). However, the nearby genes *FEN1* and *FADS3* are significant in this analysis as well, as they are also influenced by the selected haplotype. Additional lines of evidence are therefore necessary to understand which gene in a peak is the cause of the selection, and which are side-effects.

We focused on the 4 peaks that have significant genes in both 1 kG and HGDP (the HLA locus, *ACO2*, *KHK*, and *KAT8*). The HLA region is another well-established locus, but the other three are novel. *ACO2* and *KHK* are both the sole genes in their peaks that show up in both analyses, so are the most likely candidates in each. *ACO2* is a mitochondrial gene that plays an important role in the TCA cycle (Gruer *et al.* 1997), and is predicted to be relatively downregulated in Europeans, and upregulated in Peruvians from Lima (PEL) and other Native American populations. Unlike for *FADS1*, these predictions are the result of multiple SNP effects, although dominated by the allele frequencies of rs5758389 (Fig. 4c). *KHK* is a gene responsible for catabolizing dietary fructose (Bonthron *et al.* 1994), and is predicted to be downregulated in Asian populations (JPT, CHS, CDX, KHV, GIH, PJL, STU). While each of these genes was relatively isolated, the *KAT8* peak had 7 genes that replicated between 1 kG and HGDP (*KAT8*, *ZNF668*, *ITGAM*, *STX1B*, *SNF646*, *SBK1*, and *SULT1A2*). A closer look shows that this peak, in fact, represents two independent signals (Supplementary Fig. S12), with *SBK1* and *SULT1A2* showing strong predicted differences in PEL, and the other 5 genes showing predicted differences among Asian populations. Each has at least one potential candidate for selection; *ITGAM* is an integrin that is part of the innate immune system (Ramírez-Bello *et al.* 2019), while *SULT1A2* is important for metabolizing hormones, drugs, and other xenobiotic compounds (Glatt *et al.* 2001). Both are involved in responding to the environment, and are, therefore, the most likely to be subject to population-specific selection.

In contrast, the effect size-permuted *P*-values did not show a tendency to form peaks. Only 23 genes were significant in 1 kG, and none of these replicated in HGDP. These 23 significant genes perform a variety of different functions that are also potentially interesting in the context of population-specific selection, including the regulation of insulin secretion (*STXBP4*, the binding of HDL cholesterol (*HDLBP*), and viral replication (PPIE) (Wang *et al.* 2011). *SAMD10* is the gene that comes closest to replicating in 1 kG and HGDP ($P=2.0\times 10^{-6}$ in 1 kG, $P=1.2\times 10^{-5}$ in HGDP), and is a plasma membrane protein that is most highly expressed in the Cerebellum and in LCLs (Aguet *et al.* 2017). Compared to Europeans (the primary population used in training the JTI models), it is predicted to be upregulated in African and South Asian populations, particularly Gujarati, Indian Telugu and Sri Lankan Tamil, and downregulated in most East Asian populations (Supplementary Fig. S8).

## Discussion

In this study, we applied the $Q_{X}$ test for polygenic selection to regulatory variants identified using JTI expression imputation models to test for population-specific selection on gene regulation in 26 human populations. We identified 45 genes with significant regulatory selection. These included loci such as *FADS1*, *TLR*, and the HLA region that have been previously identified, as well as novel loci such as *KHK*, *SULT1A2*, and *ITGAM*. It was common for nearby genes to share high $Q_{X}$ scores, likely reflecting some combination of LD and shared regulatory variants. We also used a more conservative approach, which uses only the magnitude and direction of effects (conditioning on allele frequency differences). This version only has power to detect genes with coordinated changes across multiple variants and found few genes with evidence of selection. Some of the exceptions include genes associated with metabolism (*HDLBP*, *STXBP4*) and immunity (*PPIE*). Despite correctly identifying some well-established examples, our gene-level $Q_{X}$ score is not highly correlated with other metrics of selection, suggesting that it captures independent information.

There are some caveats with this approach. First, we are limited to testing *cis*-eQTL identified in the predominantly European GTEx data used to train the JTI models. We, therefore, cannot test for selection on *trans*-regulatory effects, or on any population-specific eQTL that were rare or absent in GTEx. Our analysis is also potentially vulnerable to confounding due to population stratification in the gene expression data, which is difficult to correctly account for. Our gamma-controlled analysis is likely susceptible to similar problems seen in the original polygenic adaptation studies (Berg *et al.* 2019; Sohail *et al.* 2019), although how much depends on the particular gene in question. In addition, while a high $Q_{X}$ does correspond to population-level differences in predicted expression in a particular tissue, these differences are not always reflective of actual differences in gene expression. More work in diverse populations and environments will be needed to confirm which of our specific results are true changes in gene expression or merely regulatory turnover.

In addition, our gamma-corrected approach highlights the fact that nearby genes are often co-expressed and share regulatory regions (Delaneau *et al.* 2019), making it difficult to determine which gene is actively subject to selection. Calculating *P*-values by permuting the effect sizes (effectively conditioning on allele frequencies) avoids identifying multiple genes in a region, but also severely limits power. While our approach does allow us to identify genes influenced by potentially selected regulatory haplotypes, this is analogous to the issues with overlapping eQTL and GWAS studies (reviewed by Cano-Gamez and Trynka 2020). A combination of LD and shared regulatory structure means signals often encompass multiple genes, and the tissue- and context-specificity of gene regulation means that our study, while genome-wide, is not exhaustive. Further lines of evidence will be required to disentangle these associations. Another caveat is that if a large proportion of genes had experienced directional selection, then including all genes when we fit the gamma distribution might be overly conservative. Another approach would be to generate a null distribution from only the middle of the distribution (Whitlock 2015). This, however, would not change the ordering of the genes and in the absence of a strong prior on the proportion of genes under selection, we decided to take the more conservative approach.

Despite these caveats, we do confirm several known instances of selection. In the case of *FADS1*, our method correctly identifies the known regulatory haplotype, and correctly predicts the direction of differences in expression between populations (Ameur *et al.* 2012; Mathieson and Mathieson 2018). The *TLR* locus is the site of a putative case of adaptive Neanderthal introgression (Quach *et al.* 2016), and our results suggest that this haplotype alters expression of all three genes in the locus. We identified several novel signals for genes involved in pathways that are likely to influence fitness in different environments. *KHK*, *ACO2*, *HDLBP*, *STXBP4*, and *SULT1A2* are all genes involved in various aspects of metabolism, whether directly diet-related or further downstream, while *PPIE* and *ITGAM* are both involved in innate immunity. We highlighted these peaks due to their occurrence in both the 1 kG and HGDP analyses; however, it should be noted that the two analyses are not directly comparable. Due to sample size limitations, while we were able to test specific populations in 1 kG, for HGDP we aggregated populations so that we were effectively testing only for differences at a continental level. It might be possible to ameliorate this issue by further adapting our approach to use a PCA-based $Q_{X}$ statistic like those described in Berg *et al.* (2019) and Josephs *et al.* (2019) rather than categorical populations.

As gene expression data become more available in other species, it will be informative to see whether the same pattern holds true in other species, particularly those with evidence of more local adaptation. Our approach would be most useful in outbred populations, since in inbred lines one can simply measure expression in each line and does not need to predict it. While this study is focused on humans, and used effect sizes modeled in multiple tissues, these are not a requirement. Our approach can be applied to any population with eQTL-level data. Regulatory models could be trained with JTI if multiple tissues are available or in a single-tissue framework (e.g. Gamazon *et al.* 2015), or simply created by clumping or pruning eQTL summary statistics.

Overall, our work suggests that strong, coordinated, population-specific selection on regulatory variation across multiple haplotypes is relatively rare among human genes and that patterns of variation in *cis*-regulation of gene expression across populations are largely explained by genetic drift. While it is possible that recent selection acts on regulatory variants we do not consider here (e.g. via *trans* effects), it is also possible that population-specific selection is not particularly common at strengths we are currently able to detect. Finally, our approach demonstrates that biologically informed tests for selection can contribute orthogonal information to those based around LD patterns or other information, and therefore could be integrated with the results of other selection scans to increase interpretability.

## Supplementary Material

iyad060_Supplementary_Data

## Data Availability

$Q_{X}$ scores and *P*-values are available as supplementary files with the manuscript, and all other data are previously published and publicly available. Scripts for parsing data files and running analyses are available from https://github.com/colbrall/gene_regulation_selection. Supplemental material is available at *GENETICS* online.
