# Supplementary material for: A gene-level test for directional selection on gene expression
Source: Genetics. 2023 Apr 10;224(2):iyad060. doi: 10.1093/genetics/iyad060 (PMC10213495; doi:10.1093/genetics/iyad060)
Supplement: iyad060_Supplementary_Data [file iyad060_supplementary_data.zip › Supplemental_Figures_GENETICS-2023-306070.pdf]

Supplementary Figures

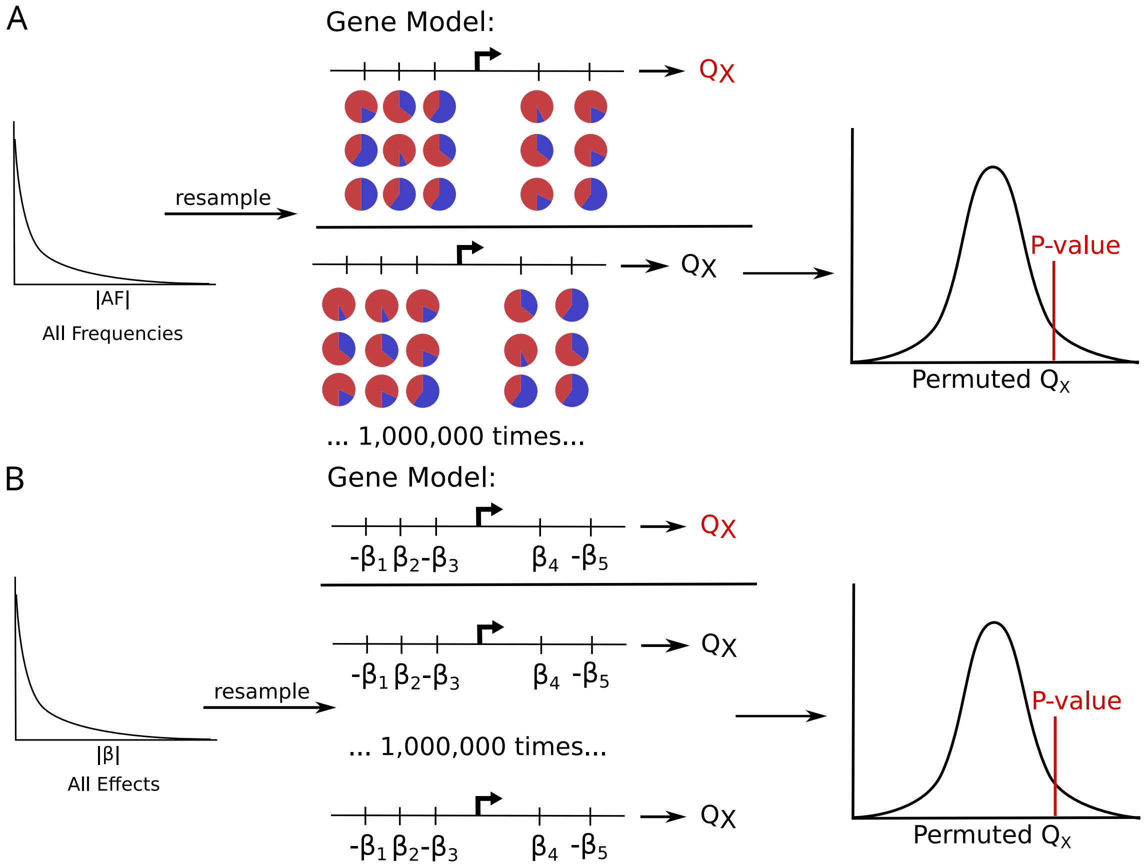

Figure S1: Schematics describing the two permutations schemes we tried.
A) One option is to calculate an empirical P-value by permuting the variant allele frequencies across a model. B) The other option is to instead permute the effect sizes of the variants, while holding effect direction constant. In both, we resampled 100,000 times for each gene, extending to 1,000,000 for those with $P < 10^{-4}$.

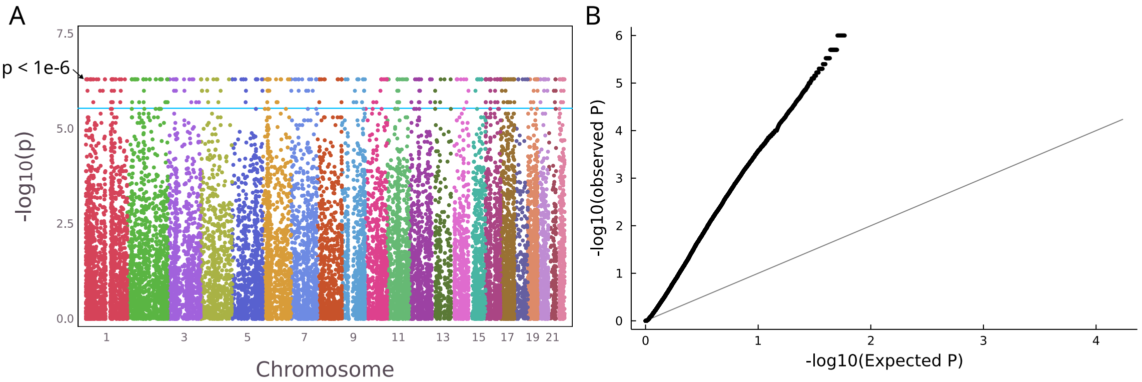

Figure S2: P-values for the Best Model protein-coding genes calculated by permuting the allele frequencies of the model variants.
A) Manhattan plot; the maximum of $-log_{10}(P)$ is where $P < 1{,}000{,}000$. B) a QQ plot of the P-values, excluding those for which $P < 1{,}000{,}000$.

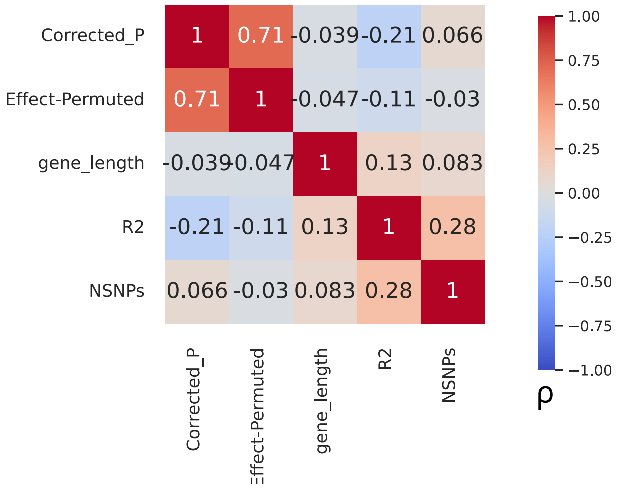

Figure S3: Q_X is correlated with technical aspects of JTI models.
Pairwise Spearman rank correlations between gamma-corrected P-values and effect size permutation P-values with various technical variables. R^2 and $NSNPs$ refer to the training performance of the Best JTI models used to calculate Q_X.

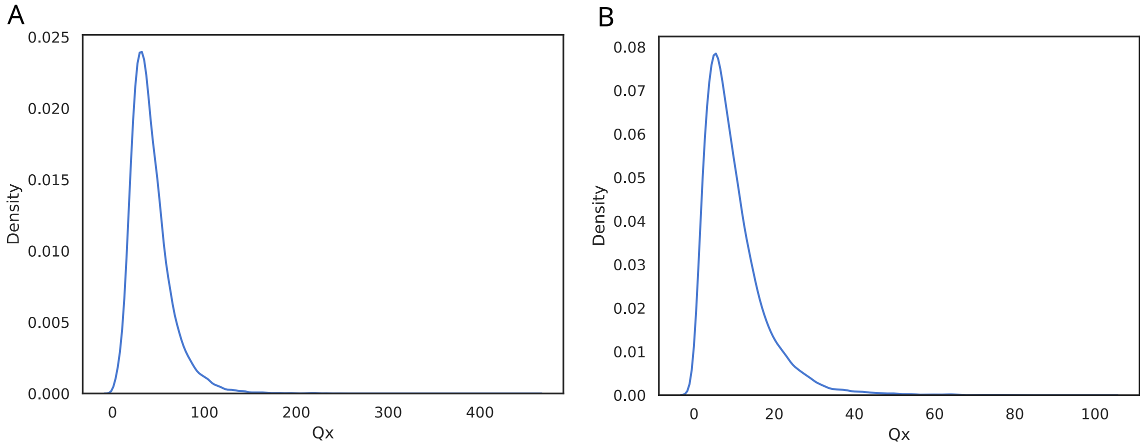

Figure S4: Distributions of Q_X scores for the Best Models of all protein-coding genes in A) 26 1kG B) 7 HGDP populations.

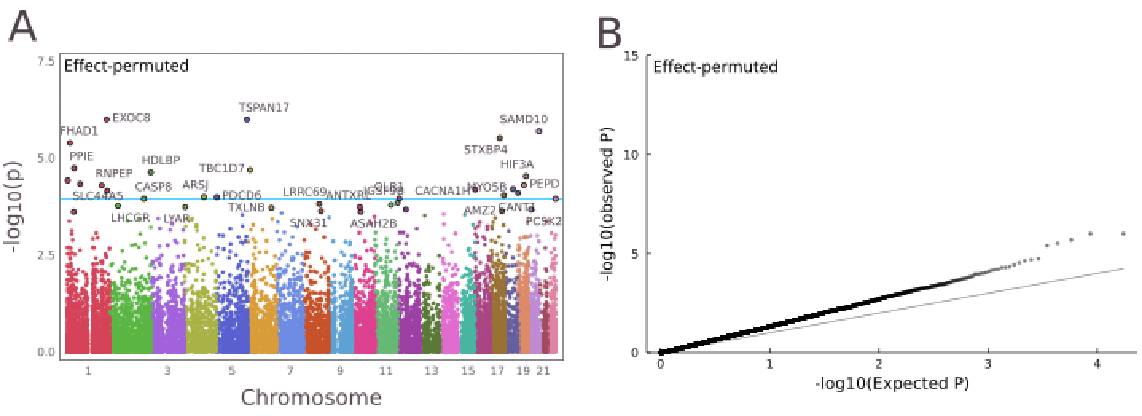

Figure S5: Effect-Permuted P-values
A) Manhattan and B) QQ plots for effect size permuted P-values (Methods). The blue line in each Manhattan plot corresponds to the threshold where FDR $<$ 0.1.

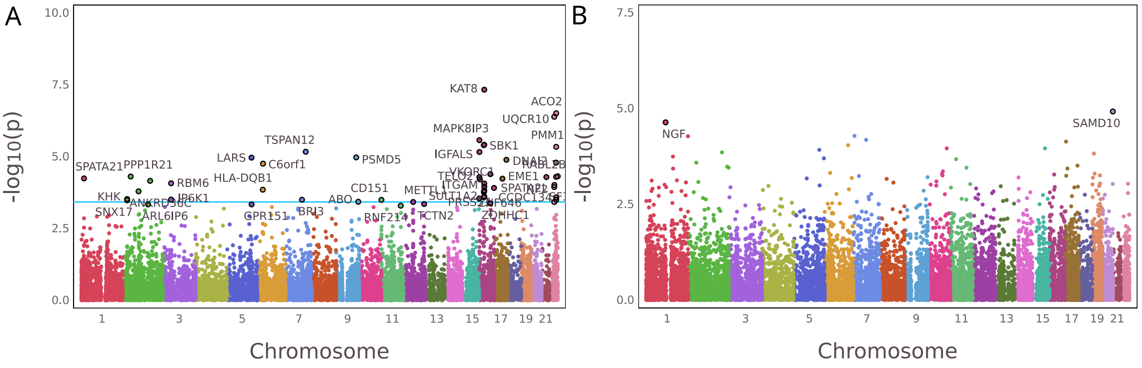

Figure S6: HGDP analysis.
Manhattan plots of A) gamma-corrected P-values and B) effect size permuted P-values for Q_X calculated in HGDP. Blue line corresponds to FDR $<$ 0.1.

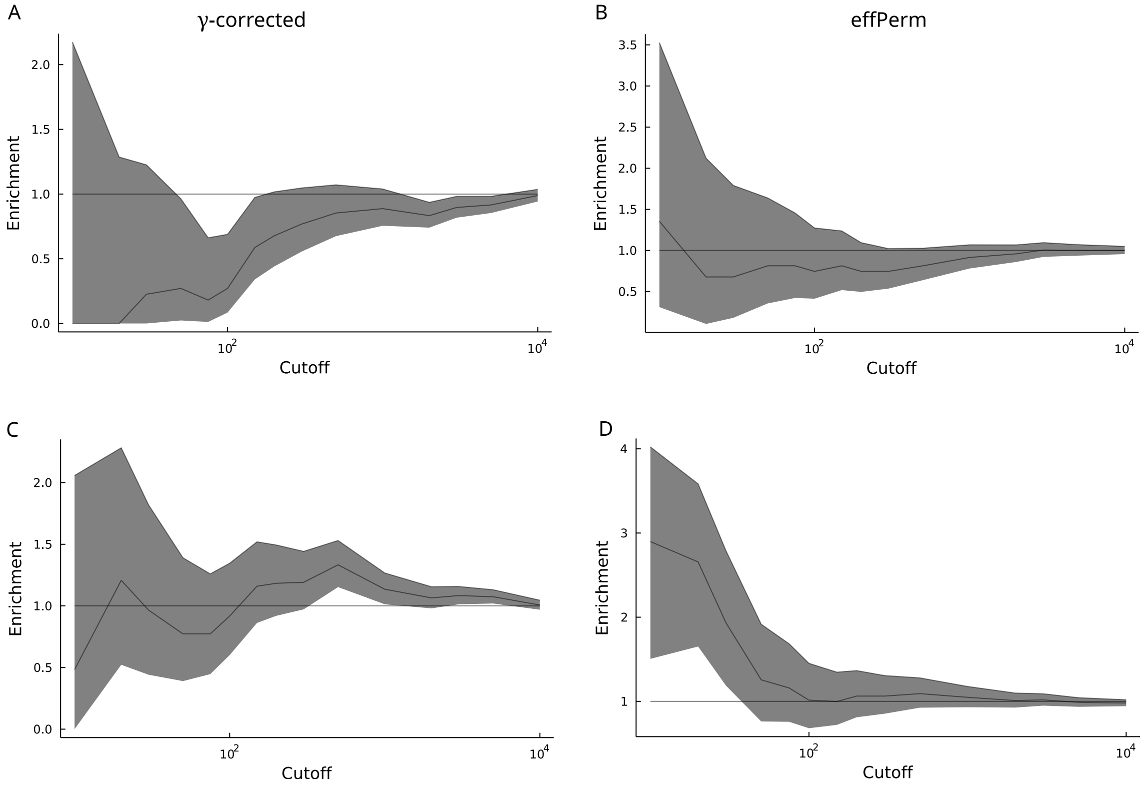

Figure S7: Predicted regulation for genes with $FDR < 0.05$ using gamma-corrected and effect size permuted P-values.
A) and B) LoF intolerant, C) and D) Housekeeping genes.

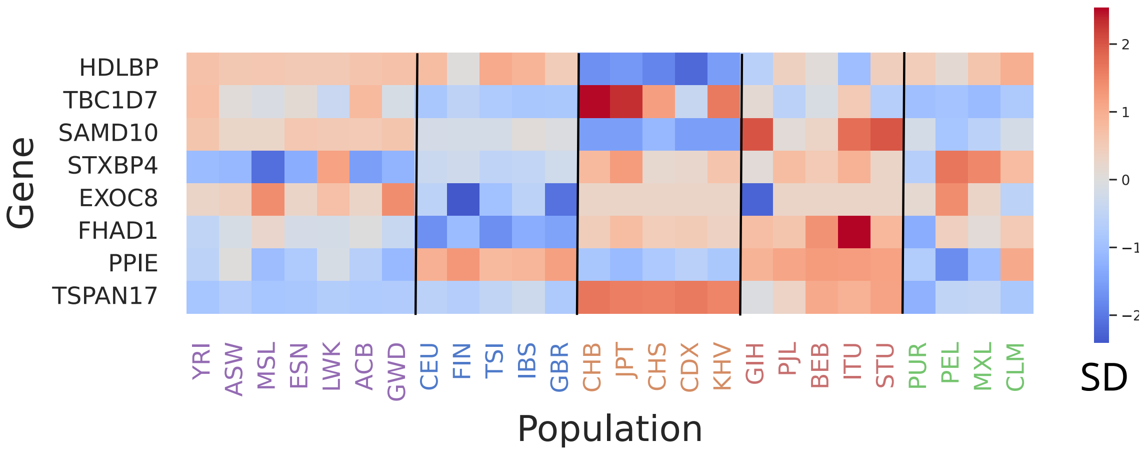

Figure S8: Predicted expression for genes with $FDR < 0.05$ using effect size permuted P-values.
Each square is coloured by the median predicted expression for that gene in that population, and is standardized across the row.

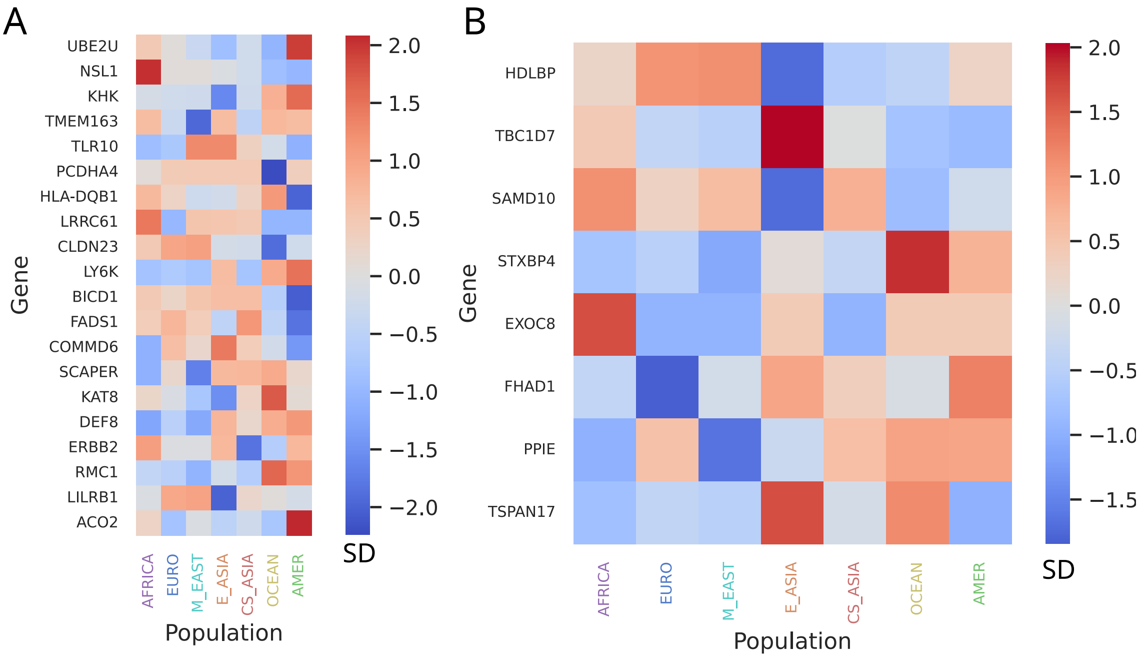

Figure S9: HGDP Predicted expression patterns largely agree with 1kG across populations.
Heatmap of median predicted expression in each HGDP population A) the top genes in each 'peak' based on the gamma-corrected P-values, and B) genes with $FDR < 0.05$ for the effPerm P-values in 1kG. For display purposes, values are standardized across populations for each gene.

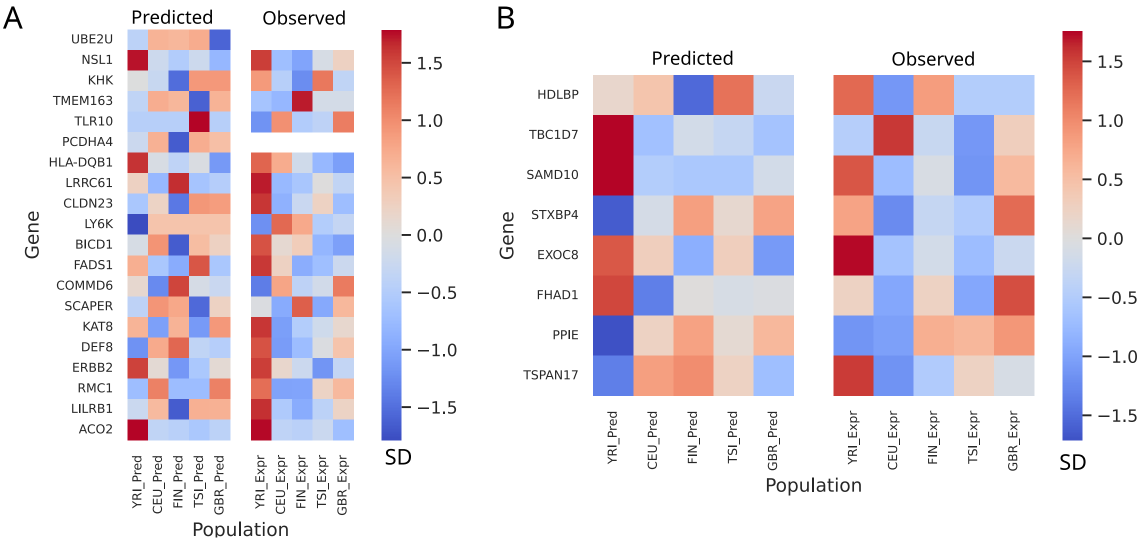

Figure S10: Predicted expression pattern does not necessarily correlate with observed expression in LCLs.
Heatmaps of median predicted and observed expression in each 1kG population for A) the top genes in each 'peak' based on the gamma-corrected P-values ($P = 0.241$), and B) genes with $FDR < 0.05$ for the effPerm P-values ($P = 0.717$). For display purposes, values are standardized across populations for each gene.

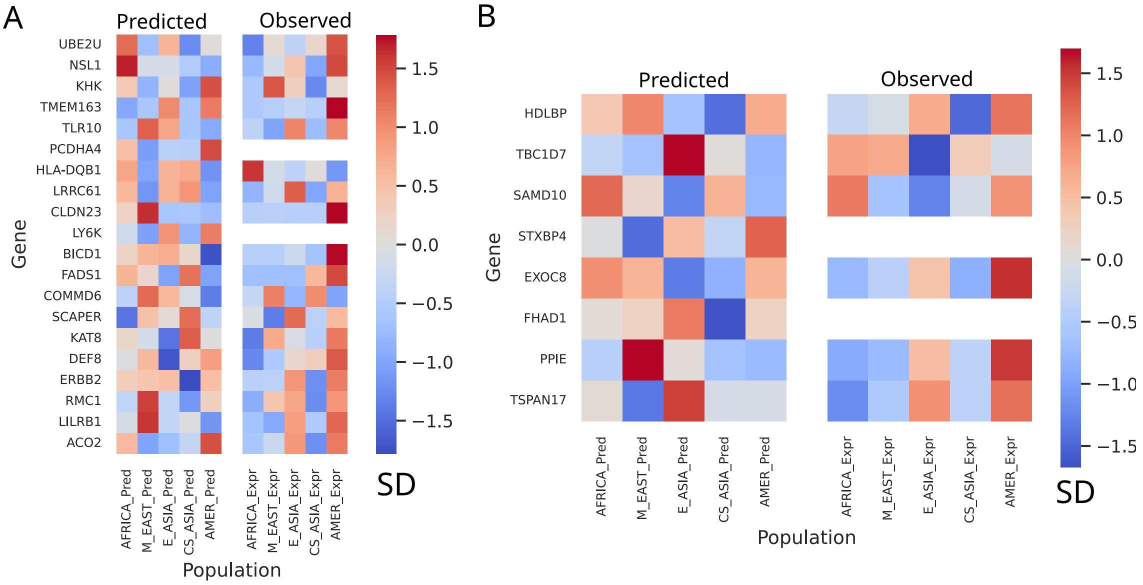

Figure S11: Predicted expression pattern does not necessarily correlate with observed expression in LCLs.
Heatmaps of median predicted and observed expression in each HGDP population for A) the top genes in each 'peak' based on the gamma-corrected P-values ($P = 0.612$), and B) genes with $FDR < 0.05$ for the effPerm P-values ($P = 0.697$). For display purposes, values are standardized across populations for each gene.

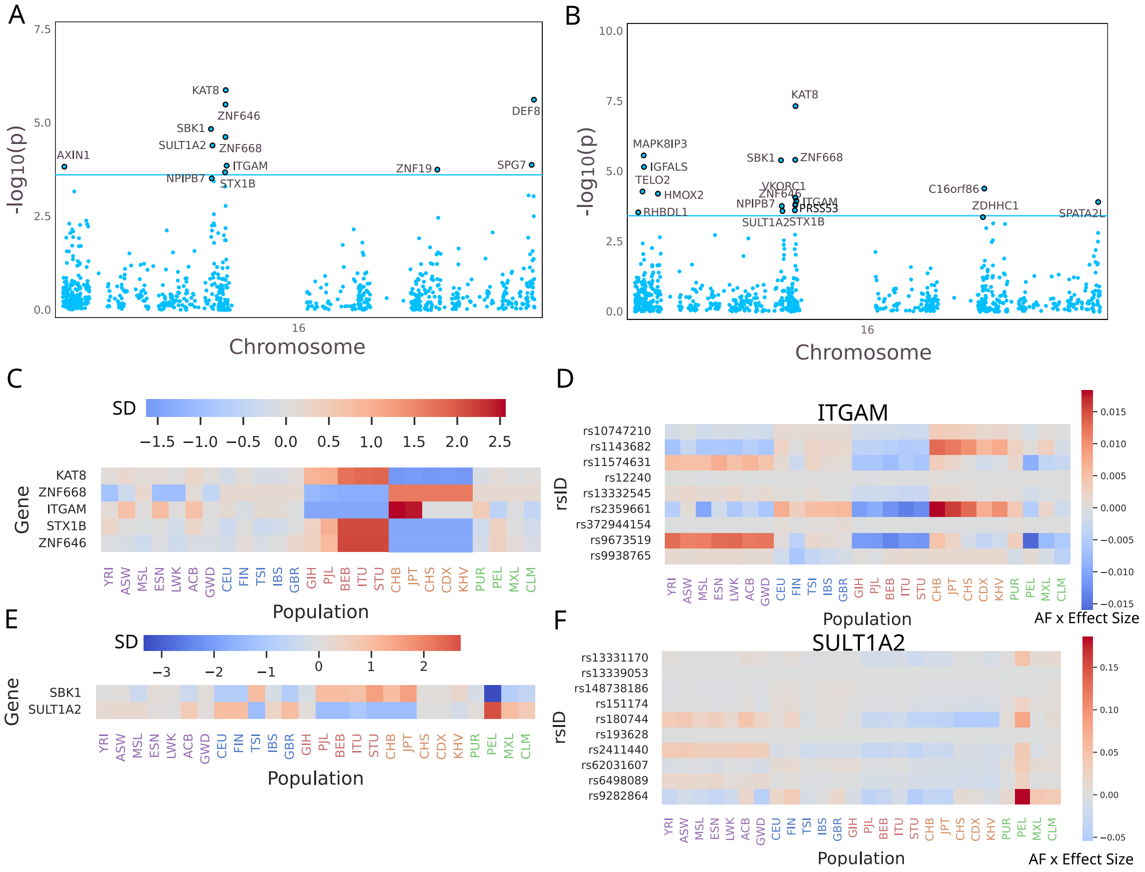

Figure S12: the *KAT8* peak is actually 2 independent signals.
Zoomed Manhattan plots of chr16 for A) 1kG and B) HGDP. C) Median Predicted expression for the 5 replicating genes near *KAT8*; D) Heatmap of the product of JTI effect size times effect allele frequency for variants in the *ITGAM* model. E) Median predicted expression for SBK1 and SULT1A2, which replicated in 1kG and HGDP, and F) Heatmap of the product of JTI effect size times effect allele frequency for variants in the *SULT1A2* model.
